# Refractory Toxic Shock-Like Syndrome from* Streptococcus dysgalactiae *ssp.* equisimilis* and Intravenous Immunoglobulin as Salvage Therapy: A Case Series

**DOI:** 10.1155/2016/2547645

**Published:** 2016-08-15

**Authors:** Marjan Islam, Dennis Karter, Jerry Altshuler, Diana Altshuler, David Schwartz, Gianluca Torregrossa

**Affiliations:** ^1^Department of Medicine, Mount Sinai Beth Israel, New York, NY 10003, USA; ^2^Department of Pharmacy, Mount Sinai Beth Israel, New York, NY 10003, USA; ^3^Department of Pharmacy, NYU Langone Medical Center, New York, NY 10016, USA; ^4^Department of Medicine, NYU Langone Medical Center, New York, NY 10016, USA; ^5^NYU School of Medicine, New York, NY 10016, USA; ^6^Department of Cardiac Surgery, Mount Sinai Beth Israel, New York, NY 10003, USA

## Abstract

Infections from* Streptococcus dysgalactiae* ssp.* equisimilis* (SDSE) can cause a wide variety of infections, ranging from mild cellulitis to invasive disease, such as endocarditis and streptococcal toxic shock-like syndrome (TSLS). Despite prompt and appropriate antibiotics, mortality rates associated with shock have remained exceedingly high, prompting the need for adjunctive therapy. IVIG has been proposed as a possible adjunct, given its ability to neutralize a wide variety of superantigens and modulate a dysregulated inflammatory response. We present the first reported cases of successful IVIG therapy for reversing shock in the treatment of SDSE TSLS.

## 1. Introduction

*Streptococcus dysgalactiae *ssp.* equisimilis* (SDSE) are gram-positive *β*-hemolytic group C and G streptococci that commonly colonize the human respiratory, gastrointestinal, and female genital tracts. Epidemiologically and pathologically, this species is very similar to Group A streptococcus, capable of causing infections ranging from mild cellulitis or pharyngitis, to life-threatening invasive disease such as necrotizing fasciitis, meningitis, endocarditis, and septic shock. It has rarely been reported to cause streptococcal toxic shock-like syndrome (TSLS), with some strains found to produce streptococcal exotoxins [1]. We present what we believe to be the first 2 reported cases of successful intravenous immunoglobulin (IVIG) therapy for adult refractory SDSE TSLS.

## 2. Case Report 1

An 82-year-old male with history of heart failure, atrial fibrillation, diabetes, and multiple prior admissions for cellulitis presented to the ED with right lower-extremity erythema with associated altered mental status, fever of 101.8°F, hypotension (72/40 mmHg), and tachycardia (146 bpm). He was admitted to the MICU for septic shock from presumed cellulitis, with initial labs significant for a lactate of 4.5 mmol/L, acute kidney injury (SCr 1.9 mg/dL), leukocytosis (white blood cell count 17 k/*μ*L, 88% neutrophils), and a procalcitonin of 38.64 ng/mL. He was requiring 26 mcg/min of norepinephrine for refractory hypotension, and vancomycin with piperacillin/tazobactam for cellulitis was initiated.

On day 2, his blood cultures grew SDSE, and vasopressin (0.04 U/min) and dobutamine were added to maintain cardiac output due to a presumed sepsis-induced myocardial depression. Despite hemodynamic support, the patient remained in shock, raising concern for development of a refractory TSLS. Surgery was consulted for potential necrotizing fasciitis and IVIG (Gamunex®-C) (1 g/kg day 1, 0.5 g/kg days 2-3) and clindamycin were initiated.

The following evening, the patient's vasopressor requirements had lessened, eventually titrated off completely over the following 24 hours. His lactate had cleared, and he did not require any surgical intervention. He was narrowed to penicillin on day 3 and transferred out of the MICU the following day. Workup for a source was inconclusive, with an abdominal CT scan showing no fluid collections or abscess. He was deemed stable for discharge on day 8.

## 3. Case Report 2

A 37-year-old male with history of coarctation of the aorta presented to the ED with fevers and diaphoresis for 2 weeks. He was initially treated for flu-like symptoms but developed rigors, abdominal pain, and jaundice, prompting him to return to our institution. On admission, he was febrile to 101°F, thrombocytopenic (platelet count 15 k/*μ*L), with leukocytosis (white blood cell count 17.5 k/*μ*L, 96% neutrophils), and with acute renal failure (SCr 1.79 mg/dL). He was started on broad spectrum antibiotics, which were subsequently narrowed to ceftriaxone (a rash developed with penicillin) and clindamycin after sensitivities revealed penicillin-susceptible SDSE (penicillin MIC < 0.06). Gentamicin was later added in the setting of persistent fevers and tachycardia. A transesophageal echocardiogram (TEE) revealed a 1.6 cm mobile tricuspid valve vegetation and a possible abscess at the aortic root after initial transthoracic echocardiogram (TTE) was inconclusive. His admission EKG showed a new 1st degree AV block.

By day 9, his heart block progressed to type II second-degree AV block, with periods of complete heart block. With concern for infectious spread to the conduction system and a perivalvular abscess, he was taken emergently to the OR for radical debridement of the aortic roots, aortomitral curtain, and interatrial septum. He underwent an extensive bovine patch repair of the aortomitral curtain, interatrial septum, and pericardium. He also underwent enlargement of the aortic annulus and roots and placement of a mechanical aortic valve and permanent pacemaker. Pathology from the aortic valve revealed acute inflammatory cells with gram-positive cocci. Postoperatively, the patient remained febrile, and antimicrobials were transiently broadened. On day 14, a TTE revealed a new mitral valve vegetation, with a perforated anterior leaflet and severe mitral regurgitation.

On day 26, repeat TEE revealed progression of mitral valve endocarditis, with a flail anterior leaflet with a second perforation, mandating surgical intervention. On day 28, the patient underwent repeat sternotomy with debridement of the mitral valve endocarditis and extensive reconstruction of the cardiac skeleton, requiring a repeat aortic and mitral valve replacement. Postoperatively, he became hypotensive and progressed into a refractory vasoplegic shock. He was given 2 doses of methylene blue and started on epinephrine (5 mcg/min), norepinephrine (19 mcg/min), and vasopressin (0.1 U/min). Despite adequate vasopressors, he remained vasoplegic, and IVIG was attempted as salvage therapy. He underwent 2 days of 100 g (1 g/kg adjust body weight) IVIG (Gamunex-C), which allowed for titration off vasopressor support over the following 2 days.

An extensive infectious workup followed to identify other potential causes for a possible culture-negative endocarditis, including serology for* B. henselae*,* B. quintana*,* Brucella, Coccidioides, Q Fever, *and* Rocky Mountain Spotted Fever*, and PCR for* Tropheryma whipplei*, all of which returned negative. On day 46, the patient underwent a final TTE, which showed no valvular vegetation. He was deemed stable for discharge, with follow-up in the cardiac surgery clinic.

## 4. Discussion

SDSE belong to a group of pyogenic streptococci, often referred to as *β*-hemolytic streptococci. While they were considered nonpathogenic for years, recent population-based studies have revealed invasive SDSE to have a similar disease profile as invasive* Streptococcus pyogenes *[1]. SDSE primarily present as skin and soft tissue infections, though invasive forms may present as osteomyelitis, pulmonary or intra-abdominal abscesses, meningitis, endocarditis, or necrotizing fasciitis. Septic shock and multisystem organ failure may result from TSLS. While injection drug users and immunosuppression pose increased risk, previously healthy individuals can develop severe infections as well [2].

Molecular studies have shown SDSE to display nearly identical virulence factors as* S. pyogenes*, though the etiology behind the emergence of more human-invasive strains remains undetermined. The principal mechanism appears to be translocation of mobile DNA elements into bacterial genomes by bacteriophages [1]. Indeed certain superantigen genes such as* speA, speC, *and* speM* have been found in SDSE strains nearly identical to those in* S. pyogenes*. Further, SDSE have consistently displayed the M protein common to* S. pyogenes*, conferring resistance to phagocytosis. In addition,* S. pyogenes* and SDSE share adhesion virulence factors such as fibronectin and plasminogen binding proteins, allowing for colonization of epithelium and invasion into the bloodstream [1].

Streptococcal TSLS is the most severe manifestation of invasive disease from streptococci, with case-mortality rates reported as high as 81% [3]. The pathogenesis behind SDSE-mediated shock likely involves the release of superantigens known as streptococcal pyrogenic exotoxins (SPEs). SPEs activate T-cell receptor molecules that directly interact with the MHC class II on antigen-presenting cells, leading to massive T-cell proliferation and a cytokine storm [2]. The effects of such a large influx of cytokines can precipitate severe vasoplegia and hemodynamic collapse, conferring the mortality seen in streptococcal TSLS [4].

SDSE remains nearly universally susceptible to penicillin and other *β*-lactam agents [1]. Clinical investigations have identified the need for an adjunctive therapy however, as high mortality rates persist despite prompt antimicrobial therapy. Since many streptococcal superantigens contribute to the pathogenesis of invasive streptococcal infections, IVIG has been suggested as the plausible adjunct, given its ability to modulate the inflammatory response elicited by virulence factors and counteract a wide variety of superantigens simultaneously [5]. It is postulated that only individuals who lack neutralizing antibodies to the putative virulence factors, such as the SPEs or M-protein, develop invasive streptococcal infections and TSLS [6]. Indeed studies have demonstrated patients with bacteremia and TSLS lacked antibodies directed against* speB*, suggesting passive immunization of patients lacking neutralizing antibodies may modify the course of this toxin-mediated disease [7].

Prior reports demonstrated IVIG's capability to block in vitro T-cell activation of staphylococcal and streptococcal superantigens. Emerging evidence has also suggested IVIG may contain superantigen-neutralizing antibodies [8]. Multiple case reports have demonstrated improved clinical outcomes in patients with TSLS who have received IVIG [9–11], while larger clinical series have supported its use. Kaul et al. demonstrated a higher 30-day survival in patients with TSLS who received IVIG compared to controls (67% versus 34%, *p* = 0.02), demonstrating an odds ratio for survival of 8.1 (CI 1.6–45, *p* = <0.01) [8]. Further, they demonstrated reduction in bacterial mitogenicity and T-cell production of IL-6 and TNF-*α* in patients who received IVIG [8]. Darenberg et al. compared IVIG therapy to placebo in patients with streptococcal TSLS and demonstrated a 3.6-fold lower mortality rate in the IVIG group, though the study was underpowered (*p* = 0.3) [12]. The IVIG group also had significantly lower sepsis-related organ failure assessment scores on days 2 (*p* = 0.02) and 3 (*p* = 0.04) compared to placebo, while also demonstrating increased plasma neutralizing activity against superantigens expressed by autologous isolates (*p* = 0.03) [12].

Upon revisiting the cases presented, it is plausible that our patients lacked the appropriate antibodies necessary to eradicate a putative virulence factor, leading to an invasive SDSE infection and ultimately succumbing into a refractory TSLS. Administration of IVIG likely helped modulate their profoundly dysregulated inflammatory responses, neutralizing the virulent super-antigens expressed by SDSE and allowing for hemodynamics to normalize. The successful use of IVIG for refractory TSLS demonstrates the utility of this novel adjunctive therapy, while highlighting the changing epidemiology and pathogenicity of SDSE.

## Additional Points

The information in this paper was not presented in any meetings at the time of submission.
